# The complete mitochondrial genome of *Sarcophaga caerulescens* (Diptera: Sarcophagidae)

**DOI:** 10.1080/23802359.2022.2035279

**Published:** 2022-03-13

**Authors:** Shujuan Wang, Zhiyun Pi, Yanjie Shang, Xiangyan Zhang, Changquan Zhang, Yadong Guo, Jifeng Cai

**Affiliations:** Department of Forensic Science, School of Basic Medical Sciences, Central South University, Changsha, Hunan, China

**Keywords:** Mitochondrial genome, *Sarcophaga caerulescens*, Phylogenetic analysis

## Abstract

*Sarcophaga caerulescens* (Zetterstedt 1838) (Diptera: Sarcophagidae) belongs to Sarcophagidae, which is closely associated with human life in ecological habits and has a clear environmental preference. *Sarcophaga caerulescens* can be better correlated with migration and postmortem interval (PMI) inference in forensic practice. In this study, we reported the complete mitochondrial genome (mitogenome) of *S. caerulescens*. The length of this mitogenome was 15,720 bp in total (GenBank accession No. MW551788), containing 13 protein-coding genes (PCGs), 2 ribosomal RNAs (rRNAs), 22 transfer RNAs (tRNAs), and a non-coding control region. Its nucleotide composition was A (39.7%), C (14.1%), G (9.4%), and T (36.9%). The phylogenetic relationships indicated that the species of *S. caerulescens* was closely related to *S. similis*. This study provides the mitochondrial data of *S. caerulescens* for further study of mitochondrial genome and enriches our understanding of the phylogenetic relationship of sarcophagid flies.

In forensic medicine, forensic entomology could provide meaningful evidence for crime scene reconstruction and postmortem interval (PMI) (Ren et al. 2018). *Sarcophaga caerulescens* (Zetterstedt 1838) was first reported to colonize human carcasses inside houses and closely associated with human life in ecological habits (Matuszewski et al. 2013). *Sarcophaga caerulescens* can be better correlated with migration and PMI inference in forensic practice.

In this study, we presented the complete mitochondrial genome of *S. caerulescens.* The adult specimens of *S. caerulescens* were first trapped by decomposing pig livers in July 2020 in Beijing city (40°22′N, 116°23′E), China. All specimens were sacrificed by freezing, and then identified based on traditional morphological features (Xue and Zhao 1996). All specimens were deposited at −80 °C in Guo’s laboratory (Department of Forensic Science, School of Basic Medical Sciences, Central South University, Changsha, Hunan, China) with a unique code (CSU20210419). Total DNA was extracted from thoracic muscle tissues using QIANamp Micro DNA Kit (QIANGEN BIOTECH Co., Ltd, Beijing, China) according to the manufacturer’s instruction. The sequencing of *S. caerulescens* mitogenome was carried out with an Illumina HiSeq 2500 Platform and then *de novo* assembly was performed using MITObim v1.9 and SOAPdenovo v2.04 (https://github.com/chrishah/MITObim and http://soap.genomics.org.cn/soapdenovo.html) (Hahn et al. 2013). Then, the preliminary annotation of all genes was determined by MITOS2 Web Server (http://mitos2.bioinf.uni-leipzig.de/index.py) (Bernt et al. 2013). The gene annotation was further verified by sequence alignment with *S. similis*. The *S. caerulescens* mitogenome has    been submitted to GenBank with accession number MW551788.

The mitogenome length of *S. caerulescens* was 15,720 bp in total, containing 13 protein-coding genes (PCGs), 2 ribosomal RNAs (rRNAs), 22 transfer RNAs (tRNAs), and a non-coding control region. The arrangement of genes was identical to that of ancestral metazoan (Cameron 2014). Its nucleotide composition was A (39.7%), C (14.1%), G (9.4%), and T (36.9%). Phylogenetic tree of *S. caerulescens* with 15 flesh flies were conducted using the Maximum-likelihood (ML) method based on 13 PCGs. ML was performed with IQ-TREE v.1.6.8 (Nguyen et al. 2015). The evolutionary model selected for ML analysis was GTR. *Chrysomya pinguis* and *Calliphora vomitoria* (Diptera: Calliphoridae) were used as outgroups (Figure 1). The phylogenetic relationships indicated that the species of *S. caerulescens* was closely related to *S. similis,* showing a clear monophyletic relationship. Accordingly, this study provides the mitochondrial data of *S. caerulescens* for further study of mitochondrial genome and enriches our understanding of the phylogenetic relationship of sarcophagid flies.

**Figure 1.**
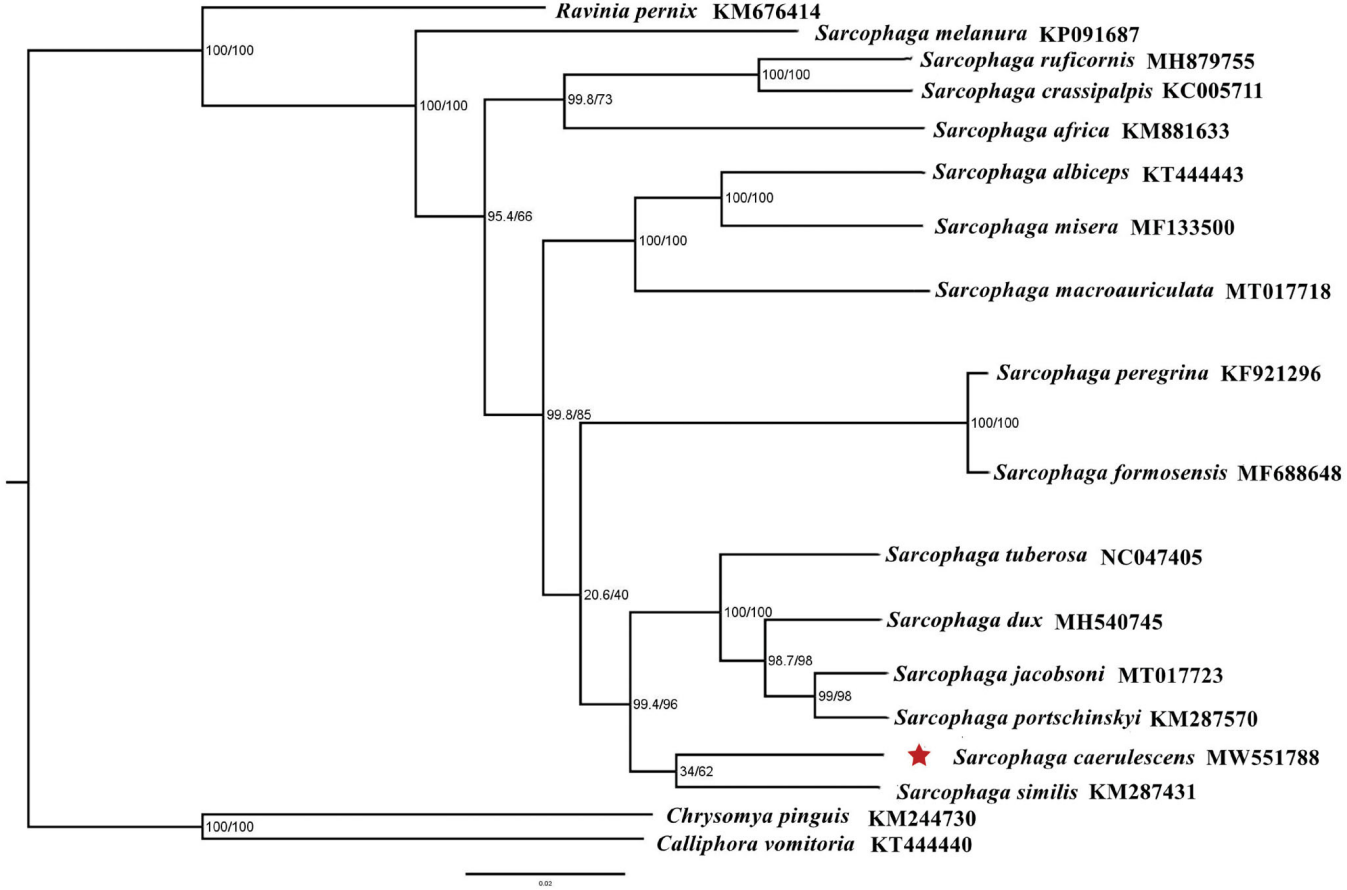
Phylogenetic trees of *Sarcophaga caerulescens* with other 15 flesh flies based on 13 protein-coding genes using the maximum-likelihood method (ML). *Chrysomya pinguis* and *Calliphora vomitoria* were selected as outgroups. Posterior probabilities/maximum-likelihood bootstrap values are shown at each node.

## Data Availability

The assembled mitochondrial genome is available on NCBI at https://www.ncbi.nlm.nih.gov/nuccore/MW551788. Associated BioProject, SRA, and BioSample accession numbers are https://www.ncbi.nlm.nih.gov/bioproject/PRJNA722817/, https://www.ncbi.nlm.nih.gov/sra/SRR14278061, and https://www.ncbi.nlm.nih.gov/biosample/SAMN18794439/, respectively. All samples were stored in Guo’s laboratory (Yadong Guo Ph.D., gdy82@126.com).
